# Breathing air to save energy – new insights into the ecophysiological role of high‐affinity [NiFe]‐hydrogenase in *Streptomyces avermitilis*

**DOI:** 10.1002/mbo3.310

**Published:** 2015-11-05

**Authors:** Quentin Liot, Philippe Constant

**Affiliations:** ^1^ INRS‐Institut Armand FrappierLavalQuébecCanada

**Keywords:** Actinobacteria, H_2_‐oxidizing bacteria, microbial seed bank, trace gas

## Abstract

The *Streptomyces avermitilis* genome encodes a putative high‐affinity [NiFe]‐hydrogenase conferring the ability to oxidize tropospheric H_2_ in mature spores. Here, we used a combination of transcriptomic and mutagenesis approaches to shed light on the potential ecophysiological role of the enzyme. First, *S. avermitilis* was either exposed to low or hydrogenase‐saturating levels of H_2_ to investigate the impact of H_2_ on spore transcriptome. In total, 1293 genes were differentially expressed, with 1127 and 166 showing lower and higher expression under elevated H_2_ concentration, respectively. High H_2_ exposure lowered the expression of the Sec protein secretion pathway and ATP‐binding cassette‐transporters, with increased expression of genes encoding proteins directing carbon metabolism toward sugar anabolism and lower expression of NADH dehydrogenase in the respiratory chain. Overall, the expression of *relA* responsible for the synthesis of the pleiotropic alarmone ppGpp decreased upon elevated H_2_ exposure, which likely explained the reduced expression of antibiotic synthesis and stress response genes. Finally, deletion of *hhySL* genes resulted in a loss of H_2_ uptake activity and a dramatic loss of viability in spores. We propose that H_2_ is restricted to support the seed bank of *Streptomyces* under a unique survival–mixotrophic energy mode and discuss important ecological implications of this finding.

## Introduction

With a typical mixing ratio of 530 ppbv, H_2_ is the second most abundant reduced trace gas in the atmosphere. Fossil fuel combustion, biomass burning, and methane oxidation are the main sources of atmospheric H_2_ (Novelli et al. 1999). Soil represents the most important sink for atmospheric H_2_ and very little is known about the ecophysiology of microorganisms involved in this important ecosystem service (Constant et al. 2009; Ehhalt and Rohrer 2009; Greening et al. 2015). *Streptomyces* sp. PCB7 was the first isolate displaying the ability to scavenge atmospheric H_2_ (Constant et al. 2008). This metabolic versatility was then demonstrated in other streptomycetes isolates, resulting in the identification of the genes *hhySL* encoding for the small and the large subunits of the putative high‐affinity group 5 [NiFe]‐hydrogenase (Constant et al. 2010). Genes encoding for group 5 [NiFe]‐hydrogenase (HAH; High‐Affinity NiFe‐Hydrogenase) were shown unevenly distributed in *Actinobacteria*,* Proteobacteria*,* Chloroflexi*, and *Acidobacteria* (Constant et al. 2011). Other isolates possessing HAH genes such as *Rhodococcus equi* and *Mycobacterium smegmatis* were also shown to scavenge atmospheric H_2_ (Meredith et al. 2013; Greening et al. 2014a).

The high‐affinity H_2_ oxidation activity (_(app)_ *K*_m_ < 100 ppmv) is restricted to mature spores in streptomycetes (Constant et al. 2008, 2010). Considering the free energy of atmospheric H_2_ oxidation, it was proposed that actinobacteria use the energy potential of atmospheric H_2_ to supply maintenance energy, implying this enzyme in bacterial persistence (Constant et al. 2011). This is in sharp contrast with the other functional groups of [NiFe]‐hydrogenases demonstrating low affinity toward H_2_ (_(app)_ *K*_m_ >1000 ppmv). These enzymes encompass four distinct phylogenetic groups and are typically involved in energy generation, H_2_‐sensing and reductive equivalents turnover during heterotrophic or lithoautotrophic growth (Vignais and Billoud 2007). As observed in the [NiFe]‐hydrogenases belonging to groups 1–4, experimental evidences suggest different physiological role within HAH found in taxonomically diverse bacteria. The first genetic investigation of HAH was undertaken in *Ralstonia eutropha* H16, the model aerobic H_2_‐oxidizing bacterium. This strain possesses four [NiFe]‐hydrogenases: a membrane bound hydrogenase (MBH; group 1), a soluble bidirectional NAD(P)‐linked hydrogenase (SH; group 3), a H_2_‐sensing hydrogenase (RH; group 2b), and a putative HAH (group 5) that were likely acquired through lateral gene transfer from actinobacteria (Schwartz et al. 2003). It appeared that HAH was weakly expressed in *R. eutropha* and purified HAH displayed low affinity for H_2_ in the presence of artificial electron acceptor, with a *K*_m_ of approximately 3 *μ*mol/L H_2_ instead of the nanomolar range observed in high‐affinity H_2_‐oxidizing bacteria (Schäfer et al. 2013). Further investigations are necessary to investigate the physiological role and the missing features of HAH for high‐affinity H_2_ uptake activity in *R. eutropha*. On the other hand, extensive genetic investigation on the three [NiFe]‐hydrogenases present in *M. smegmatis* (groups 2a, 5, and 3b) confirmed the high‐affinity H_2_ oxidation activity of HAH, but raised the doubt that high‐affinity feature is restricted to group 5 [NiFe]‐hydrogenase since mutant strain harboring group 3 [NiFe]‐hydrogenase only also displayed high‐affinity H_2_ uptake activity (Greening et al. 2014a). It was proposed that HAH provided reductants in carbon metabolism during the exponential mixotrophic growth, while supplying survival energy in resting cells (Greening and Cook 2014; Greening et al. 2014b).

Considering the different properties of HAH found in *R. eutropha* and *M. smegmatis* and the high representation of the genes encoding this enzyme in *Streptomyces*, we have undertaken a genetic investigation on the putative HAH in *S. avermitilis*. Well‐documented high‐affinity H_2_ oxidation activity (Constant et al. 2010) and availability of a genomic cosmid library necessary for efficient gene inactivation (O‐mura et al. 2001) were the main reasons to choose this model bacterium. Using a combination of transcriptomic and genetic approaches, we demonstrate that H_2_ metabolism in *Streptomyces* is unique, being restricted to their survival and dissemination. This led us to propose the concept of survival–mixotrophic energy mode and revisit the notion that substrate affinity and concentration are the main factors determining the juxtaposition of H_2_‐oxidizing bacteria across H_2_ concentration gradients in soil.

## Material and Methods

### Microorganisms

*Streptomyces avermitilis* MA‐4680 wild‐type strain isolated from Japanese soil (Kim and Goodfellow 2002) was the model high‐affinity H_2_‐oxidizing bacterium used in this study. The strain purchased from the Leibniz Institute DSMZ – German Collection of Microorganisms and Cell Cultures (strain DSM‐46492) was cultivated on MS‐agar (Kieser et al. 2000). The strain *Escherichia coli* BW25113 harboring the lambda red recombinase system (Datsenko and Wanner 2000) was used for recombination between PCR‐amplified gene deletion cassettes and cosmids. The strain *E. coli dcm* Δ(*srl‐recA*)306::Tn10 carrying pUB307‐*aph*::Tn7 kindly provided by Prof. H. Ikeda (Kitasato University, Japan) was used for conjugation with *S. avermitilis*. All *E. coli* strains were grown on Luria–Bertani medium (more details about strains, vectors, and antibiotic selections are provided in Table S1).

### Transcriptomic analysis

Spore suspension of *S. avermitilis* wild‐type strain (approximately 10^8^ spores in 100 *μ*L) was inoculated on MS‐agar amended with 10 *μ*mol/L NiCl_2_ and incubated at 30°C. After 5 incubation days, the plates showing confluent growth of aerial mycelium were transferred into enclosed systems consisting of a bottom‐free 1000 mL glass bottle mounted on a plastic support (Fig. S1). A rubber gasket sealed the bottle at the bottom of the plastic support. The enclosed system was equipped with a two‐port septum used for headspace air circulation. The first port supplied air mixture consisting either of synthetic air (0.53 ppmv H_2_ in synthetic air, designated as ambient H_2_ incubation condition; aH_2_) or H_2_ gas mixture (500 ppmv H_2_ in synthetic air, designated as elevated H_2_ incubation condition; eH_2_), while the second port was used as a vent. The flow rate of air mixtures supplying the enclosed system was set to 40 mL min^−1^ using rotameters. The system allowed a controlled H_2_ exposure of the aerial mycelia during their differentiation into mature spores. The plates were incubated 48 h in the enclosed system. Incubation under aH_2_ or eH_2_ concentration was done using three biologically independent replicates, each replicate consisting of one plate displaying confluent growth. After incubation, mature spores were rapidly harvested with a scalpel blade, transferred into 2 mL screw‐tubes containing 100 mg of 0.2 mm diameter glass beads, 700 *μ*L of TM buffer (50 mmol/L Tris‐HCl, 20 mmol/L MgCl_2_, pH 7.0), and 35 *μ*L 20% SDS and immediately frozen in liquid N_2_. Each tube containing the harvested biomass from a unique agar plate was stored at −80°C until RNA extraction. Total RNA was extracted using a chemically assisted mechanical lysis procedure (Mettel et al. 2010). A DNase treatment was performed using TurboDNase^™^ kit (Invitrogen, Carlsbad, CA, USA). Absence of residual DNA was confirmed by running the samples on agarose gel and by the absence of PCR‐amplified 16S rRNA gene with the universal primers 27F and V3R (Chakravorty et al. 2007; Frank et al. 2008). Quantification of RNA was made using a Rotor‐Gene 6000 qPCR thermocycler (Corbett Life Science^®^, New South Wales, Australia) with a QuantiFluor^™^ RNA System (Promega, Madison, WI). Total RNA samples (200 ng in 40 *μ*L per replicate) were shipped to the technical staff of *Centre d'Innovation Génome Québec et Université McGill* for quality control, library preparation, and Illumina HiSeq 2000 PE100 sequencing. Library preparation was done with TruSeq Stranded mRNA Sample Prep Kit^™^ (Illumina, San Diego, CA) and Ribo‐Zero^™^ rRNA Removal Kit (Epicentre, Madison, WI).

Demultiplexed raw sequencing results were received in paired ends fastq file format (R1 and R2 files) without barcode. The reads were 100 bases long and displayed good quality (Qphred score mean > 30 for each base). Data were filtered with fastx_tools (http://hannonlab.cshl.edu/fastx_toolkit/) to discard low‐quality fragments (q = 20, p = 90; the reads with <90% base calls with quality above Qphred score ≥20 were removed). Next, R1 files were reverse‐complemented and merged with R2 files. The six libraries were standardized to the sequencing effort of the smallest library to avoid biases in comparative analyses introduced by the sampling depth. After quality filtering and merging, each of the six independent libraries were equalized to 48 million reads, as in the smallest library, by random subsampling using the python script “subsampler.py” (David Eccles, unpubl.). The software Rockhopper (Tjaden 2015) was next used to align and annotate the reads on the genome of *S. avermitilis*. Raw sequences were deposited to the Sequence Read Archive of the National Center for Biotechnology Information under the BioProject PRJNA288961.

### Deletion of *hhySL* genes

Complete deletion of *hhySL* genes was performed using the PCR‐targeting approach described by Gust et al. (2003) with some modifications. The cosmid CL_214_G06 part of the library used to sequence the genome of *S. avermitilis* (Ikeda et al. 2003) was selected because it included the genomic fragment encoding for the HAH flanked by more than 15 kb genomic sequence. The cosmid was transferred in *E. coli* BW25113 by electroporation for two series of PCR‐targeting gene inactivation using the lambda red recombination system, induced with 100 mmol/L arabinose. The genes *hhySL* were replaced by apramycin resistance cassette (*aac3(IV)* gene disruption cassette), resulting in cosmid cPC∆1. For this purpose, the primers B‐F and B‐R were used to amplify the *aac3(IV)* gene disruption cassette from the plasmid pIJ773 (Table S2). As *S. avermitilis* is resistant to ampicillin, *bla* gene of the cosmid cPC∆1 was replaced by kanamycin resistance cassette (*neo* gene disruption cassette), resulting in cosmid cPC∆2 (Table S1). The *neo* gene disruption cassette was PCR‐amplified from pUC4K using the primers A‐F and A‐R (Table S2). The resulting cosmid cPC∆2 was transferred in the stable non‐DNA‐methylating and conjugative *E. coli dcm* Δ(*srl‐recA*)306::Tn10 carrying pUB307‐*aph*::Tn7 by electroporation. The cosmid was mobilized to S. avermitilis by conjugation. Apramycin‐resistant S. avermitilis exconjugants incapable of growth in the presence of kanamycin were selected as potential double recombinants. Double recombination was confirmed by PCR‐targeting *hhyL* and apramycin resistance and kanamycin resistance genes (Table S2).

### H_2_ oxidation activity in spores

Mature spores of *S. avermitilis* (wild‐type and *hhySL* ^−^ strains) were harvested from confluent cultures on MS‐agar amended with 10 *μ*mol/L NiCl_2_ obtained after 7 days of incubation in the dark at 30°C. Biomass was detached by scratching and repeated pipetting of 1 mL Tris‐HCl buffer (100 mmol/L, pH 7.2) on the agar surface. The suspension was transferred in a 15 mL tube and the plate was rinsed with 2 mL Tris‐HCl buffer. The resulting spore suspension (3 mL) was next filtered on glass wool fiber and the final volume was adjusted to 10 mL Tris‐HCl buffer (Kieser et al. 2000). Spore suspension was transferred into 500 mL Gibco^®^ glass bottles fitted with gastight caps equipped with butyl septa. A defined volume of air mixture containing 525 ± 10 ppm H_2_ (GST‐Welco, Reading, PA, USA) was injected to the static headspace of the sealed bottles, resulting in a H_2_ level of 3–5 ppmv. The spore suspensions were then incubated in the dark at 30°C under agitation (300 rpm). H_2_ oxidation activity was monitored using the gas chromatographic assay described by Khdhiri et al. (2015). H_2_ concentrations were monitored during 2 days. Spore concentration in the assay was measured with MS‐agar plate enumeration technique and H_2_ oxidation rate was then expressed in amol_(H2)_ c.f.u.^−1^ h^−1^. All experiments were done with three biological independent replicates, each replicate consisting of spore suspension harvested from one plate displaying confluent growth with mature spores.

### Biomass yield and spore viability in *S. avermitilis* wild‐type and *hhySL* ^−^ mutant strains

Biomass yield was calculated as the total amount of dry cell material grown in liquid culture. Both wild‐type and mutant strains were cultivated in 25 mL Bacto^™^ tryptic soy broth (Becton, Dickinson and Company, Sparks, MD, USA) at 30°C under agitation (300 rpm) during 7 days. Biomass was next collected in 15 mL tubes, centrifuged (5000*g*, 5 min), dried 24 h at 60°C, and weighted. A spore suspension containing a defined cell concentration determined with hemocytometer (Bright Line Neubauer, Hausser scientific, Horsham, PA, USA) was used to assess the viability of the spores in wild‐type and *hhySL* ^−^ mutant strains, by agar plate enumeration. Spore suspensions in 100 mmol/L Tris‐HCl (pH 7.0) were obtained from confluent cultures on MS‐agar, after filtration on glass wool fiber (Kieser et al. 2000). The viability was expressed as the fraction of total spores determined by hemocytometer that formed c.f.u. on MS‐agar after 7 incubation days in the dark at 30°C.

### Statistical analysis

Due to the small number of independent observations (three replicates) and the non‐normal distribution of the data, comparisons of spore viability, cell‐specific H_2_ oxidation rate, and biomass yield in wild‐type and *hhySL* ^−^ mutant strains of *S. avermitilis* were done by computing the Kruskal–Wallis analysis of variance on ranks using the Tukey post hoc statistical test implemented in SigmaPlot 12.3 (Systat Software Inc., San Jose, CA, USA). Comparative transcriptomic analysis of gene expression profiles was performed using the software R (R Core Development Team, 2008) using the package NOISeqBIO (Tarazona et al. 2011). Briefly, expression levels in the raw gene expression table were standardized as a function of gene length using the algorithm RPKM (Mortazavi et al. 2008) and genes represented by a minimum threshold of 1 read per million were eliminated from the dataset. The resulting standardized gene expression table was used to compute the parameters of NOISeqBIO statistics. Two parameters were computed for each individual gene, namely the log‐ratio of the average expression values and the average difference values for the two experimental conditions. Both parameters were accompanied with their standard errors to account for biological variability and then combined to derive differential expression *Z*_i_ scores (Tarazona et al. 2011). Statistical significance of differential gene expression was assessed by computing the probability of each *Z*_i_ score to differ from the random distribution of *Z*_0_ scores. This distribution of *Z*_0_ scores was determined by 1000 permutations each consisting to randomly contrast sample labels before computing gene differential expression parameters. The resulting distribution of *Z*_0_ scores consisting of noise in differential gene expression values were distributed into 15 interval classes and a Gaussian kernel density estimation of the distribution allowed to calculate the probability that two genes are differentially expressed. Differential gene expression values were considered significant when the probability to recover the same score value in the noise distribution was ≤5%.

## Results and Discussion

Soil microorganisms are exposed to trace amounts of H_2_ diffusing from the atmosphere (0.53 ppmv) and from the soil by N_2_‐fixing nodule generating steep H_2_ concentration gradients ranging from 20,000 ppmv to trace levels within a 4.5 cm radius (La Favre and Focht 1983; Witty 1991). HAH in H_2_‐oxidizing bacteria, including the spores of *Streptomyces*, thus can be saturated with substrate in nature, potentially resulting in more energy supply in the cells (Conrad 1999). In *S. avermitilis*, _(app)_ *V*_max_ of high‐affinity H_2_ oxidation activity is reached at 500 ppmv H_2_ (Constant et al. 2010). We seek to simulate hydrogenase‐saturating and nonsaturating conditions to explore the ecophysiological role of HAH in *S. avermitilis* (wild‐type strain) using a comparative transcriptomic analysis. Confluent cultures with aerial mycelium of *S. avermitilis* were exposed to a dynamic headspace constituted of synthetic air mixture with either 0.53 or 500 ppmv H_2_ (Fig. S1), corresponding to ambient H_2_ exposure treatment (aH_2_) and elevated H_2_ exposure treatment (eH_2_). Under these conditions, aerial mycelia differentiation into mature spores was completed within 24 h. Saturation of the HAH of *S. avermitilis* with 500 ppmv H_2_ did not exert significant impact on mycelium differentiation program and biomass yield, as observed by indistinguishable spore maturation phenotypes characterized with a brownish pigmentation (data not shown) and RNA extraction yields from individual confluent MS‐agar plates that were 416 ± 191 and 413 ± 102 ng *μ*L^−1^ for aH_2_ and eH_2_ exposure treatments, respectively.

**Figure 1:**
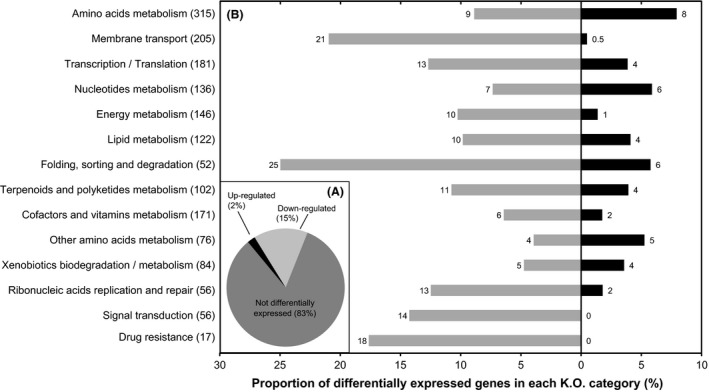
Impact of H_2_ exposure on the transcriptome of *Streptomyces avermitilis* spores. (A) Proportion of genes showing either lower, higher, or no different expression levels upon eH_2_ exposure. (B) Proportion of differentially expressed genes upon eH_2_ exposure for individual KEGG categories with grey and black bars representing the number of genes showing lower and higher expression, respectively. The total number of genes in each KO category represented along the *y*‐axis is shown between brackets. Differential expression was computed with NOISeqBIO.

Six independent libraries (3 replicates × 2 H_2_ treatments) were used for the transcriptomic analysis. In total, 92 ± 6% of the reads were successfully aligned against the genome of *S. avermitilis*, with 46 ± 2% aligned on sense protein coding genes, 2 ± 0.4% on antisense protein coding genes, 33 ± 3% on miscellaneous RNA, and 18 ± 1% on unannotated regions. The sequencing effort was proven to be sufficient, as depicted by rarefaction analysis showing transcript detection from more than 97% of the 7669 annotated genes in the *S. avermitilis* genome for each library (Fig. S2). In total, 1293 genes were differentially expressed, corresponding to 17% of the annotated genes. Among these genes, 1127 (87%) were downregulated and 166 (13%) were upregulated in eH_2_ condition (Fig. 1A). According to visual inspection of *S. avermitilis* spore morphology, H_2_ exposure did not influence the transcription profile of genes involved in cell differentiation. These genes classified into four categories encompassing spore germination, substrate mycelium transition, early sporulation, and spore maturation were not differentially expressed (Table S3). This observation confirmed that harvested biomass was at the same developmental stage and that analyzed transcriptomic profiles were representative of mature spores.

Genes showing significant differential expression in response to H_2_ exposure were classified into KEGG Orthology (KO) categories (Fig. 1B). Among the main metabolic functions proposed by KEGG, 17 comprised genes for which the expression was influenced by H_2_ exposure. Among these categories, membrane transporters, protein secretion, carbon metabolism, cellular stress, and biosynthesis of secondary metabolites were the most biologically relevant as they pointed to an unexpected HAH‐based spore self‐sufficiency in the presence of hydrogenase‐saturating H_2_ levels.

### H_2_ exposure exerts no impact on HAH genes expression profile

**Figure 2:**
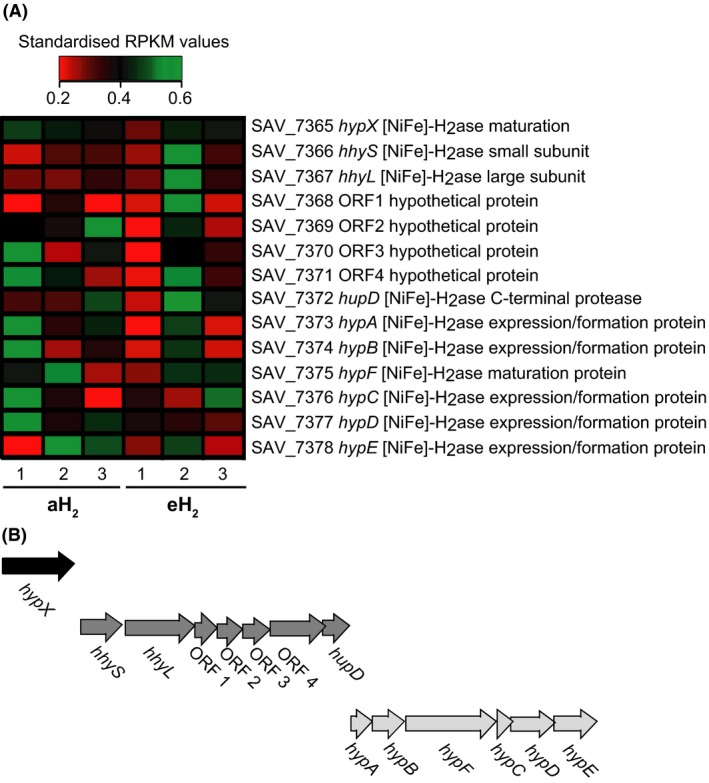
Expression of high‐affinity NiFe‐hydrogenase (HAH) operons in the spores of *Streptomyces avermitilis*. (A) The heat map represents the standardized RPKM expression level of the genes encoding the structural and auxiliary components of HAH. None of them showed significant differential expression in response to H_2_ treatments. (B) The genes encoding the structural and auxiliary components of HAH constitute three operons, as deduced by Rockhopper.

**Figure 3:**
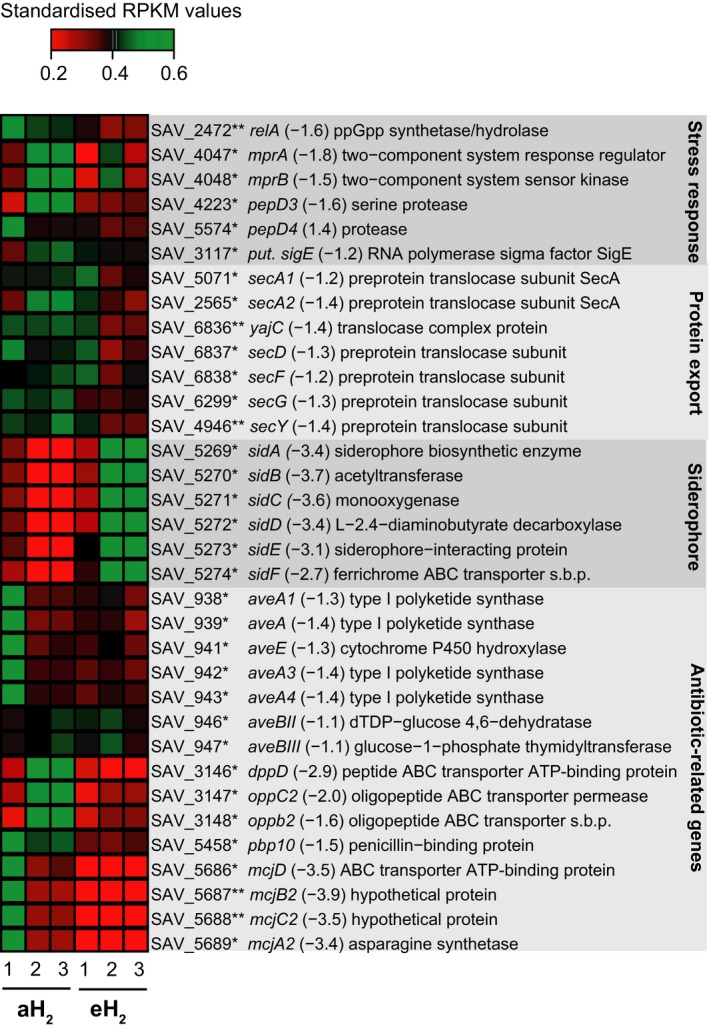
Altered expression of stress response, protein export, siderophore, and antibiotic‐related genes in the spores of *Streptomyces avermitilis*. The heat map represents the standardized RPKM expression level of the differentially expressed genes encoding for stress response, protein export, siderophore, and antibiotic‐related genes. Red and green represent lower and higher expression levels in spores upon eH_2_ exposure, respectively. The name of the gene is followed by the expression ratio (eH_2_/aH_2_) and the annotation. The asterisk represent the *P* values computed with NOISeqBIO where “*” stands for *P* ≥ 0.95 and “**” stands for *P* ≥ 0.99. The abbreviation “s.b.p.” holds for substrate‐binding protein. The whole list of differentially expressed genes is provided in Data S1.

None of the genes encoding the structural and auxiliary components of the HAH was differentially expressed upon eH_2_ exposure (Fig. 2A). This observation is in agreement with the lack of putative RH in the genome of *S. avermitilis*. Such a lack of H_2_ sensor to regulate the expression of HAH can be explained by the ubiquity of trace H_2_ in the environment. The H_2_ threshold concentration below which no oxidation activity can be detected in *S. avermitilis* is lower than 100 ppbv (Constant et al. 2010). This is far below the 530 ppbv in the global atmosphere and the 20,000 ppmv around N_2_‐fixing nodules. In contrast, low‐affinity [NiFe]‐hydrogenases found in *R. eutropha* and other knallgas bacteria display H_2_ threshold levels typically above 800 ppbv, explaining why they cannot scavenge atmospheric H_2_ (Conrad et al. 1983). These microorganisms benefit from elevated H_2_ point sources such as N_2_‐fixing nodules for mixotrophic or chemolithotrophic growth and use RH to control the expression of the auxiliary and structural components of their hydrogenases as a function of substrate availability (Lenz and Friedrich 1998; Lenz et al. 2002). However, beside this positive control by H_2_, the expression of low‐affinity hydrogenase in knallgas bacteria is activated when preferential organic carbon energy sources are absent (Friedrich 1982). In *S. avermitilis*, it is likely that intertwined regulatory systems triggered by cell differentiation program and availability of nutrients activate the expression of HAH genes restricted in mature spores (Constant et al. 2008, 2010). Although the expression level of HAH genes did not respond to H_2_ exposure, increased expression of a siderophore (*sidABCD*) and its transporter (*sidEF*) (Fig. 3) might have contributed to facilitate the maturation of the apoenzyme requiring iron in the active site of the large subunit and iron–sulfur clusters channeling electrons through the small subunit. According to their expression profile, genes encoding the structural and auxiliary components of the HAH in *S. avermitilis* are organized into one monocistronic and two polycistronic operons (Fig. 2B). The gene *hypX* was expressed as a monocistronic operon. The exact function of *hypX* is unknown (Horch et al. 2010), but its organization as a monocistronic operon can explain why this accessory gene is not always present in the genome of high‐affinity H_2_‐oxidizing bacteria (Constant et al. 2011).

### Elevated H_2_ exposure decreases the expression of external nutrient uptake

The two KO categories “folding, sorting and degradation,” and “membrane transport” were those showing the highest response upon H_2_ exposure (Fig. 1B). In total, 25% genes encompassing the category folding, sorting and degradation showed a decreased expression level in eH_2_. This response is mainly attributed to a decreased expression of the genes encoding for the general Sec protein secretion pathway. For instance, extracellular enzymes involved in the hydrolysis of recalcitrant macromolecules available in the environment (e.g., cellulose) into more labile, small molecules that can be imported into the cell possess a peptide signal recognized by the Sec secretion system. In *S. avermitilis*, 10 genes (including 2 *secA* homologs) are involved in the Sec secretion pathway. Among these, the two *secA* homologs (ATPase providing the necessary energy for protein translocation), *secGY* (two of the three genes encoding for transmembrane protein conducting channel), and *secDF* (encoding for proteins attached to the translocase complex potentially involved in the late stages of protein translocation) were differentially expressed upon eH_2_ exposure (Fig. 3). Similar response was observed in *M. smegmatis* where inactivation of HAH resulted to higher expression level of the Sec secretion system (Greening et al. 2014b). In contrast to the Sec secretion pathway, expression of the genes of the Twin Arginine Transporters (TAT) system did not respond to H_2_ exposure.

According to the lower expression of the components of the Sec secretion pathway, the expression of genes encoding for the importation of nutrients in the cell was also reduced upon eH_2_ exposure. *Streptomyces* have an important variety of permeases (e.g., 53 genes in *S. coelicolor*, 91 genes in *S. avermitilis*), mostly ATP‐binding cassette (ABC) constituting the largest KO category in *S. avermitilis* (Ikeda et al. 2003). In total, 21% (43 of the 202 genes) of these transporters were differentially expressed. With the exception of the overexpressed operon *sidABCDEF* encoding for putative iron transporter and siderophore (Ueki et al. 2009), these transporters were characterized by a lower expression level under eH_2_. Repressed transporters corresponded to permeases involved in sugars, branched‐chain amino acid, sugar alcohol, and oligopeptides transport (Data S1). Taken together, these observations point to a lower expression of the genes encoding for components participating to prospection, hydrolysis, and importation of processed nutrients in the spores when sufficient H_2_ is available to support the energy requirements.

### Influence of H_2_ exposure on primary carbon and energy metabolism

**Figure 4:**
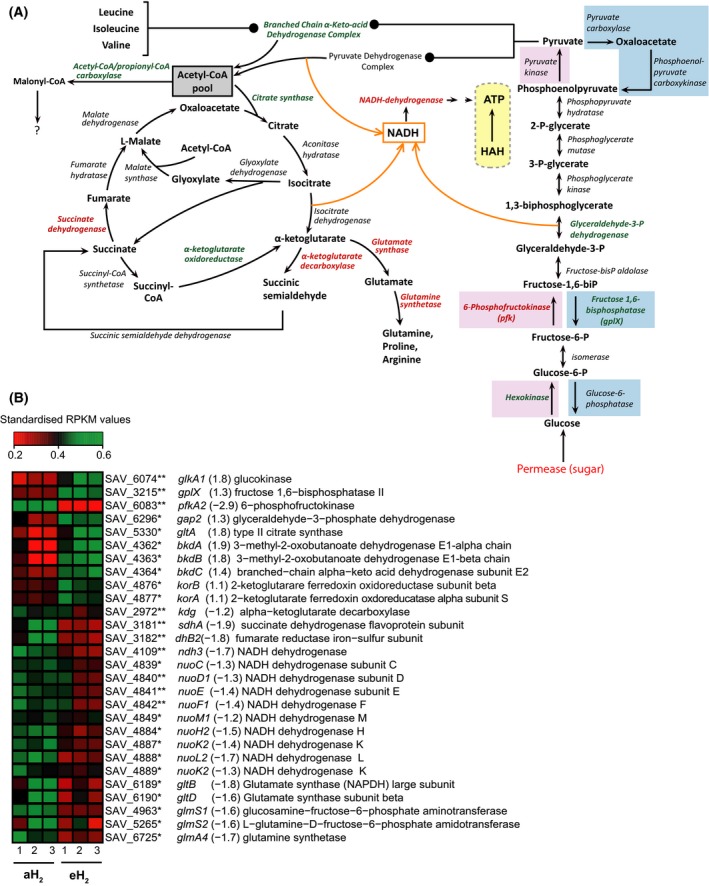
Altered expression of carbon and energy metabolic pathways in the spores of *Streptomyces avermitilis* in response to eH_2_ exposure. (A) Differential expression for the genes involved in the Embden–Meyerhof–Parnas (EMP) pathway, the TCA cycle, and the NADH dehydrogenase. The genes represented in red were downregulated while those represented in green were upregulated in the presence of eH_2_ level. The orange arrows represent different sources of NADH. The genes highlighted in the blue boxes are specific to the gluconeogenesis pathway, those highlighted in the red boxes are specific to the EMP pathway. The HAH is highlighted in a yellow box to show its potential role for ATP generation in the spores of *S. avermitilis*. (B) The heat map shows the expression level of the genes presented in (A), where red and green represent lower and higher expression level in spores upon eH_2_ exposure, respectively. The name of the gene is followed by the expression ratio (eH_2_/aH_2_) and the annotation. The asterisk represent the *P* values of the differential expression computed with NOISeqBIO where “*” stands for *P* ≥ 0.95 and “**” stands for *P* ≥ 0.99. The whole list of differentially expressed genes is provided in Data S1.

The *S. avermitilis* genome encodes four primary energy metabolism pathways: the Embden–Meyerhof–Parnas (EMP) pathway, pyruvate metabolism, the pentose phosphate pathway, and the tricarboxylic acid (TCA) cycle (Wu et al. 2005). High H_2_ exposure resulted to significant influence on EMP and TCA cycle gene expression (Fig. 4). The EMP pathway utilized to convert glucose into pyruvate and energy shares seven enzymes with the gluconeogenesis pathway. The activity of some key enzymes can indicate which direction between EMP pathway (catabolism) and gluconeogenesis (anabolism) is favored in the cell. For instance, the phosphofructokinase (Pfk) catalyses the conversion of fructose 6‐phosphate to fructose 1,6‐bisphosphate. Using ATP for substrate‐level phosphorylation, this reaction is unidirectional and represents a limiting step in EMP pathway. The reverse reaction converting fructose 1,6‐bisphosphate to fructose 6‐phosphate in the gluconeogenesis pathway is catalyzed by the fructose‐1,6‐biphosphatase (GlpX). In the presence of eH_2_, *pfkA2* showed a reduced expression level, while the gene *glpX* followed the opposite trend (Fig. 4). Even though the enzyme Pfk is subjected to allosteric regulation in the cell (Fenton et al. 2003), these observations suggest preferential activation of gluconeogenesis instead of EMP pathway in the spores exposed to eH_2_.

In addition to the oxidative conversion of pyruvate into acetyl‐CoA in the EMP pathway, acetyl‐CoA can be synthesized through the catabolism of branched‐chain amino acids. Elevated H_2_ exposure increased the expression of the branched‐chain *α*‐keto‐acid dehydrogenase complex involved in this reaction, suggesting that catabolism of leucine, isoleucine, and valine for the production of acetyl‐coA was favored to supply both TCA cycle for energy generation as well as fatty acids and polyketide biosynthesis pathways (Fig. 4A). *Streptomyces avermitilis* is characterized by an alternative TCA pathway where the oxidative and reductive pathways differ by two enzymes: the canonical *α*‐ketoglutarate dehydrogenase (Kdh) is absent and replaced by two enzymes: the *α*‐ketoglutarate decarboxylase (Kgd) and the succinic semialdehyde dehydrogenase. These enzymes bridging oxidative and reductive TCA half‐cycles were first identified and biochemically characterized in *M. tuberculosis* (Tian et al. 2005). The expression of gene *kgd* decreased under eH_2_, while the expression of the *α*‐ketoglutarate oxidoreductase (Kgor) showed the inverse trend (Fig. 4). Combined with the decreased expression of succinate dehydrogenase and glutamate synthase/synthetase, these observations suggest that glyoxylate shunt of the TCA pathway was favored when the spores were exposed to eH_2_. This pathway is responsible for anaplerotic reactions replenishing the intermediate of TCA cycle, allowing growth on acetate and fatty acids. Transcriptomic profile did not show evidence for increased catabolism of storage lipid in spores exposed to eH_2_. With the exception of a well‐documented depletion of glycogen deposits coordinated with accumulation of trehalose during sporogenesis (Brana et al. 1986; Rueda et al. 2001), very little is known about the primary metabolism of streptomycetes spores. Our data suggest that a combination of stored energy sources and exogenous organic carbon supply the energy requirements in the spores, but further investigations will be necessary to assess carbon flux distribution and metabolism.

In addition to generate ATP, the EMP pathway and TCA cycle generate reduced nicotinamide adenine dinucleotide (NADH) used as an electron carrier supplying the respiratory chain through the NADH dehydrogenase. Under eH_2_ exposure, the expression of 10 of the 28 NADH dehydrogenase subunits genes decreased (Fig. 4). The physiological electron acceptor of HAH in *S. avermitilis* is unknown but decreased expression of NADH dehydrogenase suggests that HAH participates to the generation of proton motive force for ATP generation in the spores. Even though NADH dehydrogenase genes did not show increased expression in the HAH deletion mutant of *M. smegmatis*, the activity was mainly observed in the membrane fraction (Greening et al. 2014a,b), which is an argument in favor of a link of HAH with the respiratory chain for ATP generation.

### Elevated H_2_ exposure resulted to a lower cellular stress

During translation of mRNA into protein, a nutrient deficiency results in blockage of the ribosome when deacylated tRNA binds in the ribosomal A‐site. This causes a stringent stress response induced by the RelA protein that generates alarmone ppGpp (guanosine tetraphosphate). The alarmone ppGpp changes the affinity of the RNA polymerase for some promoters (including the ribosomal) in favor of those that encode stress response genes. In the case of *Streptomyces*, the alarmone informs a state of famine, which induces cell differentiation accompanied by the production of secondary metabolites (Kang et al. 1998; Hoyt and Jones 1999). Spores exposed to eH_2_ showed decreased expression of *relA*, responsible for the production of the alarmone ppGpp (Fig. 3). This observation supports the hypothesis of self‐sufficiency presented in the previous sections describing the impact of H_2_ on Sec protein secretion pathway, sugar transport and carbon metabolism. Lower expression of *relA* might explain the decreased expression of avermectin biosynthesis genes (7), beta‐lactamases linked genes (3) and genes involved in the biosynthesis of the antimicrobial peptide microcin under eH_2_ exposure (Fig. 3). Furthermore, the penicillin‐binding protein *pbp10* gene and transporter genes conferring beta‐lactam resistance (*dppD*,* oppC2, oppB2*) showed lower expression level under eH_2_ (Fig. 3). This is consistent with the decreased expression of the putative sigma factor *σ*E and the putative components of the stress detection system including *mprAB* involved in signal transduction and the protease *pepD* that are expressed when the cell envelope loses its integrity in response to external cues such as membrane‐targeting antibiotics, including beta‐lactams. The energy supplied by HAH leading to a decreased dependence on exogenous organic substrate thus appeared to reduce stress related to nutrient starvation through the stringent response, resulting to a decrease of secondary metabolite biosynthesis.

### Extending gene expression profile response through genetic inactivation of the HAH

**Figure 5:**
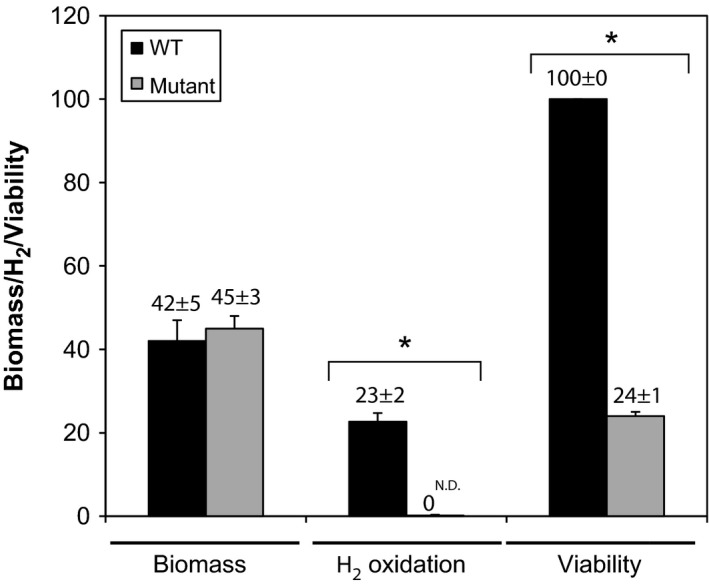
Impact of HAH inactivation on the phenotype of *Streptomyces avermitilis*. Cell‐specific H_2_ oxidation rate in spores (expressed in amol c.f.u.^−1^ h^−1^), viability of spores (expressed in %), and biomass yield of vegetative cultures in TSB broth (expressed in mg mL^−1^) of wild‐type and *hhySL* ^−^ strains are presented. The symbol “*”denotes significant difference in H_2_ oxidation activity and viability between wild‐type and *hhySL* ^−^ mutant strains (ANOVA,* P* > 0.95). The term “N.D.” denotes the absence of detectable H_2_ oxidation activity in the spores of *hhySL* ^−^ mutant strain. All experiments were done with three independent replicates, with the exception of spores viability in wild‐type strain (two independent replicates).

Our previous observations suggest that a lack of HAH would influence fitness in the spores of *S. avermitilis*. This hypothesis was tested by the deletion of *hhySL* genes in the model bacterium. The deletion of *hhySL* through double recombination was confirmed by antibiotic resistance phenotypes and PCR (Fig. S3A). There was no significant difference in the biomass production yield between wild‐type and *hhySL* ^−^ strains after vegetative growth in TSB broth (Fig. 5), further highlighting the fact that HAH is mainly expressed and active in spores and does not support mixotrophic growth. This observation is in sharp contrast with *M. smegmatis* for which inactivation of HAH resulted in a decreased biomass yield characteristic to classical mixotrophic growth reported in knallgas bacteria (Berney and Cook 2010). Growth of the mutant strain on MS‐agar led to mature dark brown‐colored spores, visually indistinguishable from the wild‐type spores (Fig. S3B). As expected, spores of wild‐type strain displayed high‐affinity H_2_ oxidation activity, while no significant H_2_ uptake rate was detected in the mutant strain (Fig. 5). According to the hypothesis of HAH‐based self‐sufficiency model derived from theoretical energy yield of H_2_ oxidation (Constant et al. 2011) and the transcriptomic analysis presented in this study, spores of the mutant strain showed a drastic loss of viability, with 4.2 folds less regeneration than the wild‐type strain (Fig. 5).

### Survival–mixotrophy as a new paradigm for the ecology of H_2_‐oxidizing bacteria

H_2_ concentration is currently perceived as a driving force behind the definition of the ecological niche of aerobic H_2_‐oxidizing bacteria in soil (La Favre and Focht 1983). Indeed, soil microbial communities are exposed to two different diffuse sources of H_2_, namely the atmosphere and legume nodules generating H_2_ as obligate by‐product of N_2_‐fixation (Hoffman et al. 2009). This results in microniches encompassing 20,000 ppmv to subatmospheric levels (<0.530 ppmv) of H_2_ (Hunt et al. 1988; Rasche and Arp 1989; Witty 1991; Constant et al. 2009). Two complementary experimental evidences suggest that juxtaposition of H_2_‐oxidizing bacteria in soil is defined by H_2_ concentration gradients. On one hand, H_2_ oxidation activity in soil is characterized by a biphasic kinetics explained by the occurrence of two subpopulations of H_2_‐oxidizing bacteria demonstrating either low or high‐affinity for H_2_ (Häring and Conrad 1994). On the other hand, low‐affinity H_2_‐oxidizing bacteria were shown to be enriched and/or activated following elevated H_2_ soil exposure (La Favre and Focht 1983; Popelier et al. 1985; Dong and Layzell 2001). Both substrate affinity and threshold concentration below which hydrogenase cannot scavenge H_2_ thus limit the beneficial effect of this energy source on the distribution of H_2_‐oxidizing bacteria in soil. However, the notion of two extreme subpopulations defined by their differential affinity toward H_2_ was questioned after the observation of intermediate _(app)_ *K*_m_ values in soil (Schuler and Conrad 1991) and *Streptomyces* isolates (Constant et al. 2010), suggesting the occurrence of a continuum of H_2_‐oxidizing bacteria subpopulations in nature.

Taken together, our results suggest that the mechanism explaining the impact of H_2_ concentration gradients on the structure of microbial communities is beyond the limitation of substrate affinity and threshold concentration. Indeed, we demonstrate for the first time that HAH homologs in different taxonomic groups of bacteria are characterized by distinct ecophysiological roles. For instance, the HAH is unevenly distributed in a number of *Actinobacteria* species encompassing *Mycobacterium* and *Streptomyces* (Constant et al. 2010). In *M. smegmatis*, H_2_ was used both as an energy source for mixotrophic growth and to supply the respiratory chain during long‐term persistence (Berney and Cook 2010; Greening et al. 2014b). In contrast, H_2_ uptake activity was restricted to mature spores in *S. avermitilis*, where H_2_ appeared to reduce the dependence on carbon energy sources for persistence and dissemination under a mixotrophic‐based survival mode. These observations suggest that diffuse sources of H_2_ will induce different response in soil *Mycobacterium* and *Streptomyces* subpopulations. It is expected that the biomass of H_2_‐oxidizing *Mycobacterium* would increase directly upon elevated H_2_ exposure owing to their mixotrophic growth. On the other hand, H_2_ exposure would favor the persistence rather than the growth of the spores of H_2_‐oxidizing *Streptomyces*, resulting to a delayed response of their biomass to H_2_ exposure. Under this assumption, substrate affinity is not the principal factor influencing the distribution of H_2_‐oxidizing bacteria. Considering the reduced or increased expression of secondary metabolites and extracellular proteins secretion in *S. avermitilis* in response to differential H_2_ exposure as well as the complex nature of microbe–microbe interactions in soil, there is an imperative need to better understand the impact of H_2_ on soil microbial community structure and function (Stein et al. 2005; Zhang et al. 2009; Osborne et al. 2010). Metabolomic investigation of carbon flux in spores thriving under mixotrophic survival mode would be crucial to validate and better understand the proposed HAH‐based self‐sufficiency model in the spores of high‐affinity H_2_‐oxidizing streptomycetes.

## Conflict of Interest

None declared.

## Supporting information


**Data S1.** Excel spreadsheet showing all differentially expressed genes along with their p‐value and eH2/aH2 expression ratio.

**Figure S1.** Illustration of the dynamic microcosm chambers used for the transcriptomic analysis.
**Figure S2.** Assessment of the sequencing effort invested in the transcriptomic analysis.
**Figure S3.** (A) Confirmation of double recombination in *Streptomyces avermitilis hhySL* ^−^ by PCR. (B) Photograph of confluent cultures on MS‐agar to show the indistinguishable phenotype between wild‐type and *hhySL* ^*−*^ mutant strains.
**Table S1.** Strains and vectors utilized in this study.
**Table S2.** List of oligonucleotides utilized and their associated PCR conditions.
**Table S3.** Absence of differential expression for genes involved in four development stages of streptomycetes.
