# Supplementary material for: Breathing air to save energy – new insights into the ecophysiological role of high‐affinity [NiFe]‐hydrogenase in Streptomyces avermitilis
Source: Microbiologyopen. 2015 Nov 5;5(1):47–59. doi: 10.1002/mbo3.310 (PMC4767420; doi:10.1002/mbo3.310)
Supplement: Supplementary file 2 — Figure S1. Illustration of the dynamic microcosm chambers used for the transcriptomic analysis. Figure S2. Assessment of the sequencing effort invested in the transcriptomic analysis. Figure S3. (A) Confirmation of double recombination in Streptomyces avermitilis hhySL − by PCR. (B) Photograph of confluent cultures on MS‐agar to show the indistinguishable phenotype between wild‐type and hhySL − mutant strains. Table S1. Strains and vectors utilized in this study. Table S2. List of oligonucleotides utilized and their associated PCR conditions. Table S3. Absence of differential expression for genes involved in four development stages of streptomycetes. [file MBO3-5-047-s002.docx]

**Supplementary Information**

Table S1. Absence of differential expression for genes involved in four development stages of streptomycetes.

Table S2. Strains and vectors utilised in this study.

Table S3. List of oligonucleotides utilised and their associated PCR conditions.

Figure S1. Illustration of the dynamic microcosm chambers used for the transcriptomic analysis.

Figure S2. Assessment of the sequencing effort invested in the transcriptomic analysis.

Figure S3. (a) Confirmation of double recombination in *S. avermitilis hhySL*^-^ by PCR. (b) Photograph of confluent cultures on MS-agar to show the indistinguishable phenotype between wild type and *hhySL^-^* mutant strains.

**Table S1.** Absence of differential expression for genes involved in four development stages of streptomycetes. The genes classified into four categories encompassing (1) spore germination, (2) substrate mycelium transition, (3) early sporulation and (4) spore maturation (Chater, 1998; Flärdh and Buttner, 2009). Differential expression was computed with the package NOISeqBIO.

| **Genes** | **Category** | **Diff. Expr.** | **Description** |
| --- | --- | --- | --- |
| SAV_2630 (*whiG)* | 4 | no | The gene *whiG* encodes an RNA polymerase sigma factor and may be a key regulator of the commitment of aerial hyphae to sporulation. |
| SAV_2684 *(hupB)*  SAV_5127 *(HU1)* | 4 | no | HU protein contributes to spore nucleoid compaction and is required for the development of spore heat resistance. |
| SAV_4331 (*whiP)* | 4 | no | WhiP influences the coordination of aerial hyphal extension and septation, possibly by inhibiting cell division until the correct moment. |
| SAV_4997 (put. *whiB*) | 4 | no | The biochemical role of Wbl proteins is controversial: they might function as transcription factors or they might be disulphide reductases. |
| SAV_6294 *(whiA)* | 4 | no | WhiA constitutes, together with WhiB, a WhiG-independent converging pathway that controls sporulation in aerial hyphae. |
| SAV_2445 *(whiH)* | 4 | no | WhiH mutants have reduced spore pigmentation and make mostly undifferentiated aerial hyphae |
| SAV_4185 (*sigF*) | 3 | no | The RNA polymerase sigma factor SigF controls late development during sporulation. |
| SAV_6861 *(bldD)*  SAV_2529 *(bldD)* | 2 | no | BldD acts as a repressor of some known developmental regulatory genes : *bldN/M*, *whiG* and *sigF* in *S. coelicolor* |
| SAV_4130 *(bldC)* | 2 | no | The gene *bldC* encodes a MerR-family transcription factor that is conditionally required for aerial mycelium formation. |
| SAV_3150 *(bldKA1)*  SAV_3151 *(bldKB1)*  SAV_3152 *(bldKC1)*  SAV_3153 *(bldKD1)*  SAV_3154 *(bldKE1)* | 2 | no | The gene *bldK* encodes an oligopeptide importer implied in cellular differentiation. |
| SAV_3172 *(bldKE2)*  SAV_3173 *(bldKD2)*  SAV_3174 *(bldKC2)*  SAV_3175 *(bldKB2*  SAV_3176 *(bldKA2)* | 2 | yes (0.64)  yes (0.66)  yes (0.60)  yes (0.57)  yes (0.71) | The gene *bldK* encodes an oligopeptide importer implied in cellular differentiation. |
| SAV_5455 *(mreB1)*  SAV_5456 *(mreC)*  SAV_5457(*mreD*) | 1 | no | MreB is an actin-homologs expected to form cytoskeletal filaments under the cytoplasmic membrane. |

**Table S2.** Strains and vectors utilised in this study. The following antibiotics were added to the growth media for marker selection and plasmid maintenance (in µg ml^-1^): carbenicilin (100), apramycin (50), and kanamycin (12.5) for *E. coli* and *S. avermitilis.*

| **Strains** | **Carried vector** | **Purpose** | **Reference** |
| --- | --- | --- | --- |
| *E. coli* BW25113 | pKD46 | Carries λred system, facilitates recombination | Datsenko and Wanner 2000 |
| *E. coli* *dcm* Δ(*srl-recA*)306::Tn10 | pUB307-*aph*::Tn7 | Transfer of non-methylated vectors by conjugation in *S. avermitilis* | Kitani et al 2009 |
| *E. coli* B1 | pKD46 and CL_214_G06 | Recombination between CL_214_G06 and the PCR-amplified apramycin resistance cassette | This study |
| *E.coli* B2 | pKD46 and cPC∆1 | Recombination between cPC∆1 and the PCR-amplified *neo* gene | This study |
| *E. coli* B3 | pKD46 and cPC∆2 | Extraction of the cosmid cPC∆2 to be transferred in *E. coli* C1 by electroporation | This study |
| *E. coli* C1 | pUB307-*aph*::Tn7 and cPC∆2 | Transfer of non-methylated cPC∆2 by conjugation in *S. avermitilis* | This study |
| *S. avermitilis* MA-4680 |  | Wild type strain | Kim and Goodfellow 2002 |
| *S. avermitilis* ∆2SR | Integrated cPC∆2 | Single recombinant of cPC∆2 | This study |
| *S. avermitilis* *hhySL*^-^ | none | Double recombinant K.O. mutant of *hhySL* | This study |
|  |  |  |  |
| **Vectors** | **Gene(s) carried** | **Antibiotic resistance (host)** | **Reference** |
| CL_214_G06 | *hhySL* | Carbenicilin (*E. coli*) | Ōmura et al 2001 |
| pIJ773 | *aac3(IV)* cassette | Apramycin (*E. coli*) | Gust et al 2003 |
| pUC4K | *neo* | Kanamycin (*E. coli*) | GE HelthCare Life Science |
| cPC∆1 | *aac3(IV)* cassette | Carbenicilin and apramycin (*E. coli*) | This study |
| cPC∆2 | *aac3(IV)* cassette + *neo* | Apramycin and kanamycin (*E. coli* and suicide vector in *S. avermitilis*) | This study |

**Table S3.** List of oligonucleotides utilised and their associated PCR conditions. The oligonucleotides utilised for PCR-targeted gene replacement have 39 nt of homology (underlined) to the 5’ upstream region (forward primer) or 3’ downstream region (reverse primer) of targeted gene. PCR mixtures consisted of 1X reaction buffer (15 mM MgCl_2_), 10% Band Sharpener, 0.2 mM dNTP, 20 µM of each primer, 1.25 U Fast-Taq DNA polymerase (Feldan^®^, QC, Canada), 2 µl template DNA and nuclease-free water to obtain a final volume of 50 µL.

| **Fragment** | **Name** | **Primers** | **PCR conditions** |
| --- | --- | --- | --- |
| *neo*-in-cPC | A | A-F: 5’-AGGCACCTATCTCAGCGATCTGTCTATTTC GTTCATCGCTGAGGTCTGCCTCGTG-3’  A-R: 5’-TCAGAATGACTTGGTTGAGTACTCACCAGT CACAGAGAAAGCCACGTTGTGTCTC-3’ | 94°C for 5 min, 30 cycles of “slowdown steps” denaturing at 94°C for 30 s, annealing temperature starting at 65°C decreasing 1°C in every 3 cycles to reach a temperature of 55° (45 s at each cycle), and a elongation step of 72°C for 3 min. |
| apramycin cassette | B | B-F: 5’-GGACTTTCACCCCATACCTCCCTAGGAGGA GGCGGTCCCATGATTCCGGGGATCCGTCGACC-3’  B-R: 5’-GCTCGGCGGTCACGCGGTTCGTCGCCGATACCG CGCTCATGTAGGCTGGAGCTGCTTC-3’ | 94°C for 5 min, 30 cycles of “slowdown steps” denaturing at 94°C for 30 s, annealing temperature starting at 65°C decreasing 1°C in every 3 cycles to reach a temperature of 55° (45 s at each cycle), and a elongation step of 72°C for 3 min. |
| *neo* | C | C-F: 5’-GGCGCTTTCTCAATGCTCA-3’  C-R: 5’-GCCATCCTATGGAACTGCCT-3’ | 94˚C for 5 min, 35 cycles of 94˚C for 30 sec, 57˚C for 30 sec, 72˚C for 75 sec followed by a final elongation step at 72°C for 5 min. |
| *hhyL* | D | D-F: 5’-ATGGCATCGACGACGAAGGC-3’  D-R: 5’-TCATCCGCCCAGTCCGCTCA-3’ | 94˚C for 5 min, 35 cycles of 94˚C for 30 sec, 57°C for 30 sec, 72˚C for 90 sec followed by a final elongation step at 72°C for 5 min. |
| partial *hhyL* | E | E-F: 5’-ATCTCGGGBATCTGYGGKGACAA-3’  E-R: 5’-ATGAGRCAACATCTCYCGGGT-3’ |  |

**Figure S1.** Illustration of the dynamic microcosm chambers used for the transcriptomic analysis. (a) Schematic of dynamic microcosm chambers showing the rotameters supplying air (aH_2_ or eH_2_ level) in parallel replicated microcosms containing one Petri dish. (b) Detailed view of one microcosm chamber. The air was continuously injected in the microcosm chambers (tube 1) and evacuated by the vent (tube 2) in the top of the chamber. (c) Picture of one microcosm used in this study.


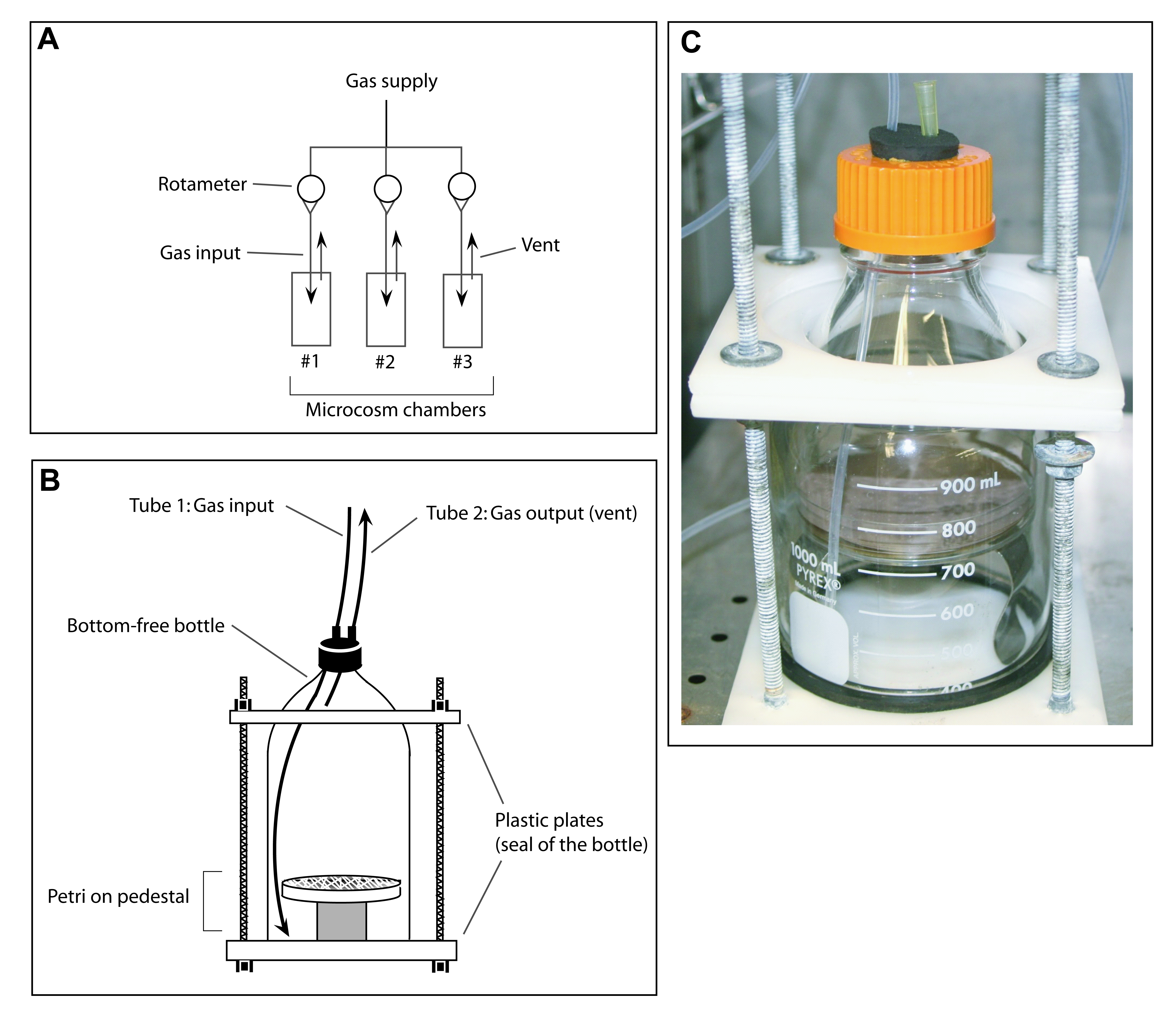


**Figure S2.** Assessment of the sequencing effort invested in the transcriptomic analysis. The rarefaction curves were computed with the function “explo.plot” implemented in the package NOISeq. The solid dots represent the actual amount of sequences in each sample, while the empty dots are simulated by NOISeq. A plateau was reached, resulting in the detection of at least 97.1% know genes in *S. avermitilis*.


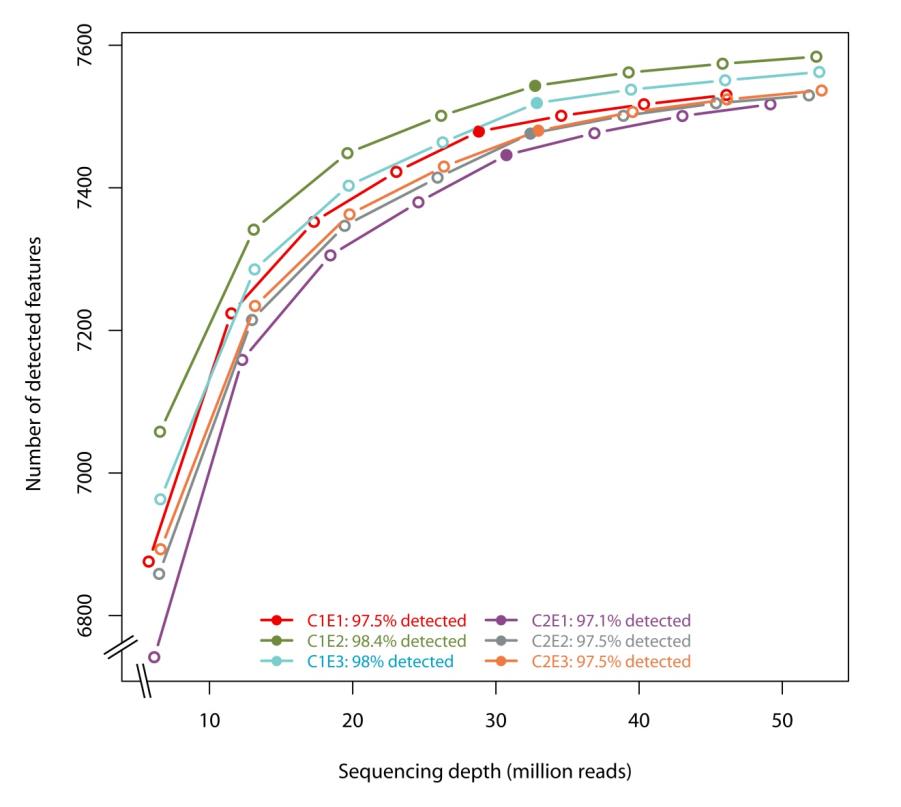


**Figure S3.** (a) Confirmation of double recombination in *S. avermitilis hhySL*^-^ by PCR. The amplified fragments were analysed by agarose gel electrophoresis as follows: lane 0: 1 kb DNA ladder (Feldan, Québec, QC, Canada), a: partial *hhyL* (primers F), b: *hhyL* (primers E), c: *aac(3)*IV cassette (primers B), d: *neo* (primers C); 1: positive control (genomic DNA of *S. avermitilis* wild type), 2: negative control, 3: genomic DNA from putative double recombinant *S. avermitilis hhySL^-^ #3* used as template*,* 4: genomic DNA from putative double recombinant *S. avermitilis hhySL^-^ #12* used as template. Double recombinant *S. avermitilis hhySL^-^ #3* was selected for this study. (b) Photograph of confluent cultures on MS-agar to show the indistinguishable phenotype between wild type (left side) and *hhySL^-^* mutant (right side) strains after 7 days of incubation in the dark at 30°C.


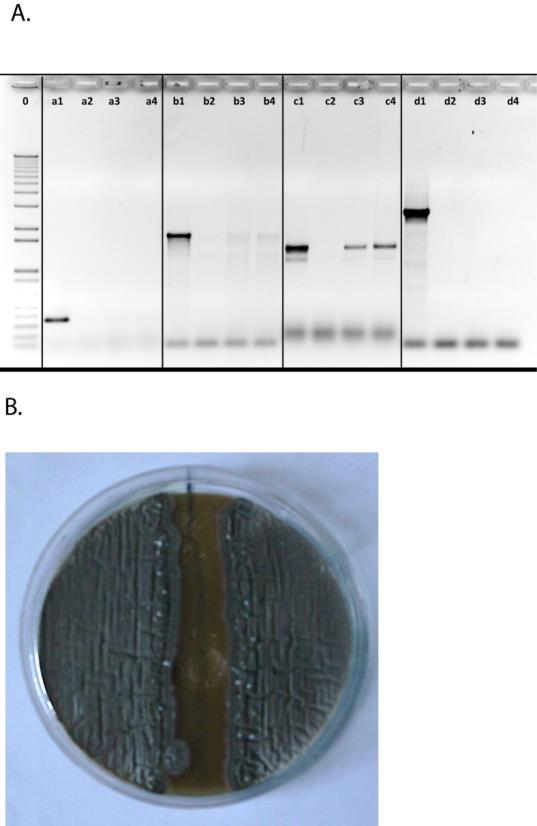


**References**

Chater KF (1998). Taking a genetic scalpel to the *Streptomyces* colony. Microbiology 144:1465-1478.

Datsenko KA, Wanner BL (2000). One-step inactivation of chromosomal genes in *Escherichia coli* K-12 using PCR products. Proc Natl Acad Sci 97:6640-6645.

Flärdh K, Buttner MJ (2009). *Streptomyces* morphogenetics: dissecting differentiation in a filamentous bacterium. Nat Rev Microbiol 7:36-49.

Gust B, Challis GL, Fowler K, Kieser T, Chater KF (2003) PCR-targeted *Streptomyces* gene replacement identifies a protein domain needed for biosynthesis of the sesquiterpene soil odor geosmin. Proc Natl Acad Sci 100:1541-1546.

Kitani S, Ikeda H, Sakamoto T, Noguchi S, Nihira T (2009). Characterization of a regulatory gene, *aveR* , for the biosynthesis of avermectin in *Streptomyces avermitilis*. Appl Microbiol Biotechnol 82:1089-1096.

Ōmura S, Ikeda H, Ishikawa J, Hanamoto A, Takahashi C, Shinose M et al (2001). Genome sequence of an industrial microorganism Streptomyces avermitilis: Deducing the ability of producing secondary metabolites. Proc Natl Acad Sci 98:12215-12220.
